# Apparatus for Simultaneous Small Angle Neutron Scattering and Steady Shear Viscosity Studies of Polymer Melts and Solutions

**DOI:** 10.6028/jres.095.003

**Published:** 1990

**Authors:** Alan I. Nakatani, Hongdoo Kim, Charles C. Han

**Affiliations:** National Institute of Standards and Technology, Gaithersburg, MD 20899

**Keywords:** deuterated polystyrene, phase separation, polymer blends, poly(vinylmethylether), small angle neutron scattering, viscosity

## Abstract

The design and construction of an apparatus for studying the simultaneous small angle neutron scattering (SANS) and steady shear viscosity behavior of polymer melts and concentrated solutions is discussed. Successful operation of the device is demonstrated on a blend of 20 weight percent deuterated polystyrene and 80 weight percent poly(vinylmethylether). The effects of shear on the critical behavior of the blend are observed in the SANS behavior as a function of temperature and shear rate and indicate shear induced mixing behavior for the range of shear rates examined. The steady shear viscosity results alone are insufficient for detecting the transition from one to two phases. The examination of shear effects in polymer blends is important for understanding the critical behavior of binary systems. Technologically, knowledge of the phase behavior of polymer blends under shear are important for the design and improvement of commercial blend processing.

## 1. Introduction

The shear behavior of mixtures near a critical point is of fundamental importance for testing theories of critical behavior. In simple binary fluid systems [1–4], a crossover from non-classical behavior to mean field behavior is expected under the influence of simple shear. Shifts in the critical point on the order of 0.001 K with the application of extremely high shear rates (thousands of reciprocal seconds) have been observed. The temperature control and range of shear rates necessary to examine these small effects in simple fluids is very difficult to achieve.

Polymer blends have displayed mean field behavior under quiescent conditions, and have demonstrated shifts in the critical point on the order of a degree to even tens of degrees with the application of much lower shear rates [5,6]. Therefore, polymer blends are much more amenable to studying the effect of shear on the critical behavior of a mean field system. However, the behavior of polymer blends with shear is not without complications. Different investigators report either shear induced mixing or shear induced demixing behavior. The variation in the results has also spawned a variety of theoretical treatments and predictions for polymer phase behavior during shear. Summaries of various work in this field are given by Rangel-Nafaile et al. [7] and Tirrell [8].

The technological importance of the effect of shear on polymer blends must also be considered. The morphology of polymer blends is directly related to processing conditions. By gaining an understanding of the phase behavior of polymer blends during processing, it may be possible to improve current commercial blends or to develop conditions for the advancement of new polymer blends.

This work will describe an apparatus for studying the simultaneous steady shear viscosity and small angle neutron scattering (SANS) behavior of polymer melts, blends, and concentrated solutions in a controlled, uniform shear geometry. Because of the small wavelength of the incident neutron radiation, concentration fluctuations in sheared-polymer systems on a length scale from a few angstroms to a few hundred angstroms may be obtained with this device. Most previous studies on the shear behavior of polymer blends utilized light-scattering techniques which are only capable of probing concentration fluctuations on the order of 1000 Å. Examination of the fluctuations on a much smaller size scale as well as determining the relationship between the size of the concentration fluctuations and the viscosity behavior is desirable for a microscopic understanding of the system. On the other hand, viscosities of the materials of interest may range from a few poise to millions of poise and phase boundaries for polymer blends range from ambient temperature to over 200 °C, which makes the torque and temperature requirements for the apparatus quite severe. With this in mind, the design and construction of the apparatus will be discussed as well as preliminary data on a blend system of deuterated polystyrene (*M*_w_ = 4.4 × 10^5^) (440K PSD) and poly(vinylmethylether) (*M*_w_ = 1.8 × 10^5^) (180K PVME).

## 2. Experimental

### 2.1 Apparatus

**Figure 1:**
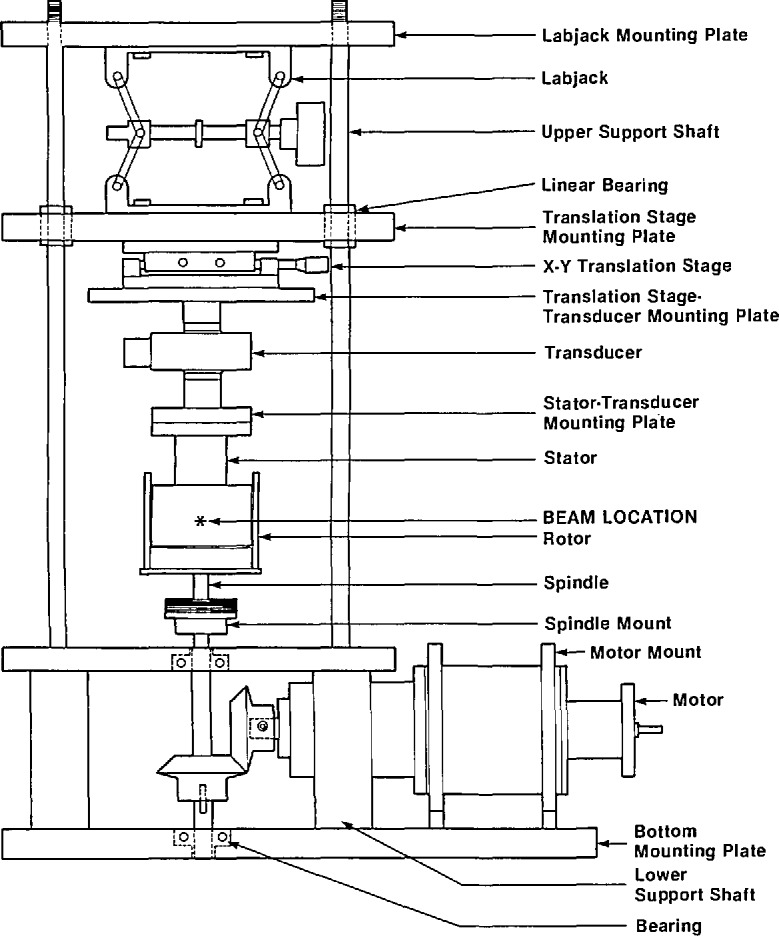
Schematic of SANS shear cell.

The SANS shear cell was designed to fit within the constraints of the SANS instrument at NIST (fig. 1). The only limitations to the apparatus are transparency to neutrons and a tabletop to beamline distance of 30.48 cm. The apparatus design is similar to that constructed by Lindner and Oberthur [9,10] for use at Institut-Laue Langevin (ILL) in Grenoble, France, with a few important differences. A high torque system with uniform rotor speeds at low shear rates was desirable for studies on concentrated solutions and melts. The SANS shear cell is of couette geometry with an inner radius of the quartz cylinder (rotor) of 4.552 cm. The quartz is bonded with epoxy to an Invar spindle to form the cup. Radial runout tolerances are all less than 0.0013 cm.

The rotor is driven through a set of miter gears by a brushless dc servo motor with an encoder feedback loop and quadrature detection. Two different motors and two different encoders are utilized. The first motor has a 50:1 harmonic speed reducer with a maximum torque of 67 N-m while the second motor has a 4.96:1 helical gear reduction box with a maximum torque of approximately 5 N-m. The two encoders have a resolution of 500 and 2500 pulses per revolution (ppr); the 2500 ppr encoder is particularly useful for extremely slow motor speeds. With this combination of motors and encoders nominal shear rates from 0.02 to 7000 s^−1^ may be attained. Viscosities as high as 1.0 × 10^6^ poise have been successfully examined in the shear cell. The upper limit to the viscosity is determined by the strength of the quartz to Invar bond of the rotor.

**Figure 2:**
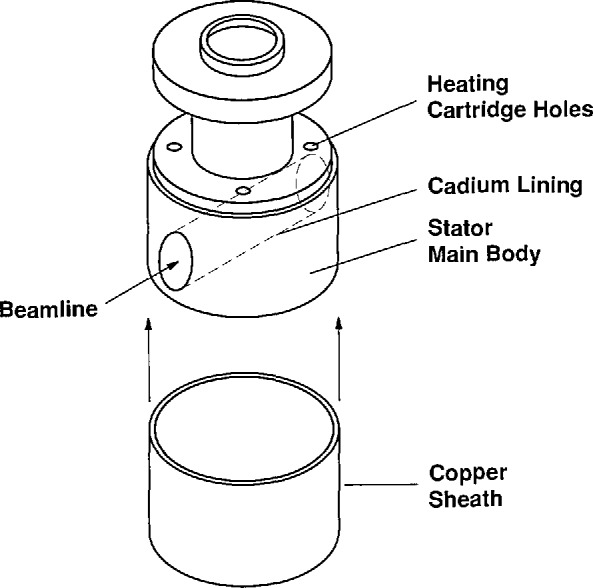
Exploded view of copper stator (drawing not to scale).

The inner cylinder of our device (stator) is unique to the apparatus. Instead of quartz, the stator is constructed from oxygen-free, pure copper. Pure copper has a low neutron cross section and is one of the few metals which can be used successfully as a window material in SANS experiments. The nominal radius of the cylinder at room temperature is 4.506 cm and the height is 5.347 cm. The cylinder has a 3.50-cm diameter hole bored in the transverse direction for the beam path. A thin sheath of copper (0.040-cm thick) is then heat shrunk over the cylinder. Thermal expansion of the cylinder provides a tight fit between the sheath and cylinder (fig. 2). Mechanically, the stator appears as a solid piece of copper; to neutrons the stator is nearly transparent. Copper was utilized instead of quartz to allow for a broader range of temperature control as well as better uniformity and accuracy. Quartz, having a very low thermal conductivity, was not deemed suitable for these purposes. Similar devices [9–11] have a temperature controlling fluid circulating through a quartz stator. Temperature control in our apparatus is achieved by use of four heater cartridges placed in the copper block with PID control of the heater power supply. Additional control is achieved by a secondary electrical heating mantle which surrounds the entire stator/rotor assembly. The control circuit is designed so that the temperature of the external mantle follows the PID controlled temperature of the copper stator. The upper operating temperature is about 170 °C. Alignment of the stator is accomplished by means of a precision x–y stage. Vertical translation of the stator is accomplished using a laboratory jack (Newport Corporation)^1^ with extra precision linear ball bushing bearings guiding the x–y translation stage mounting plate on four 3.175-cm diameter, precision-ground rods (straightness tolerance 4.26 × 10^−5^ cm/cm).

The gap between the rotor and stator is nominally 0.5 mm. The exact value was determined based on the linear thermal expansion coefficient of the copper stator. Dimensional changes of the quartz are assumed to be negligible. The bottom of the stator is a truncated 2° cone to reduce edge effects. The shear rate at the bottom is much lower than the shear rate in the gap for a given rotation rate and the contributions to the torque can be neglected.

The transducer, which is connected to the stator, is a combination torque/normal force transducer. The torque component is a static torque transducer with a load capacity of 56.5 N·m and using a digital indicator is accurate to 0.0045 N·m. Higher sensitivity may be achieved by using a chart recorder and monitoring the voltage output of the transducer directly. The thrust component of the transducer is a 2.22 × 10^3^ N capacity load cell. The purpose of this load cell is limited to monitoring thrust on the sample during loading and locating the bottom of the cup.

For a Couette geometry viscometer, the shear rate, *γ*, and the viscosity, *η*, are determined as follows [12]:

$$\begin{array}{c} \dot{\gamma }=\omega R_{1}/\left(R_{2}-R_{1}\right)\\ \eta =T\left(R_{2}-R_{1}\right)/\left(2\pi R_{1}{\;}^{3}H\omega \right) \end{array}$$

where ω is the angular velocity, *R*_2_ is the inner radius of the quartz cylinder, *R*_1_ is the radius of the copper cylinder, *T* is the torque, and *H* is the height of the copper cylinder. Two Newtonian viscosity standards were used from Cannon Instruments, S8000 (238.2 poise at 25 °C) and S30,000 (816.9 poise at 25 °C, 223.1 poise at 40 °C and 103.6 poise at 50 °C) to check the performance of the instrument.

## 2. Materials

Polystyrene-*d*_8_ (PSD) for this experiment was prepared by anionic polymerization of styrene-*d*_8_ in benzene with butyllithium as an initiator using standard techniques [3]. The PSD was characterized by gel permeation chromatography (GPC) showing *M*_w_ = 4.4 × 10^5^ and *M*_w_/*M_n_* = 1.28. Poly(vinylmethylether) (PVME) was polymerized by cationic polymerization in toluene with BF_3_-ethyl ether complex as an initiator [14]. The polymer was fractionated in heptane and the fraction utilized in these experiments had a *M*_w_ = 1.8 × l0^5^ and *M*_w_/*M*_n_ = 1.76 as determined by GPC. The blend composition was 20 weight percent PSD and 80 weight percent PVME corresponding to the critical composition for a PSD/PVME blend [15]. The sample was prepared by dissolving the polymers in toluene and then pouring the solution into the bottom of the rotor and removing the toluene by vacuum in a vacuum oven at 80 °C for 48 h.

## 3. Results

SANS results were obtained using an incident wavelength of 9 Å at the NIST reactor. Data was collected over a two-dimensional detector and corrected for background and dark current intensity due to electronic noise. Sector averages of the scattered intensity parallel and perpendicular to the flow (horizontal and vertical) were obtained in 10° sectors. Absolute intensity calibration was done with a dry silica gel as a secondary standard, calibrated in terms of a primary vanadium standard.

**Figure 3:**
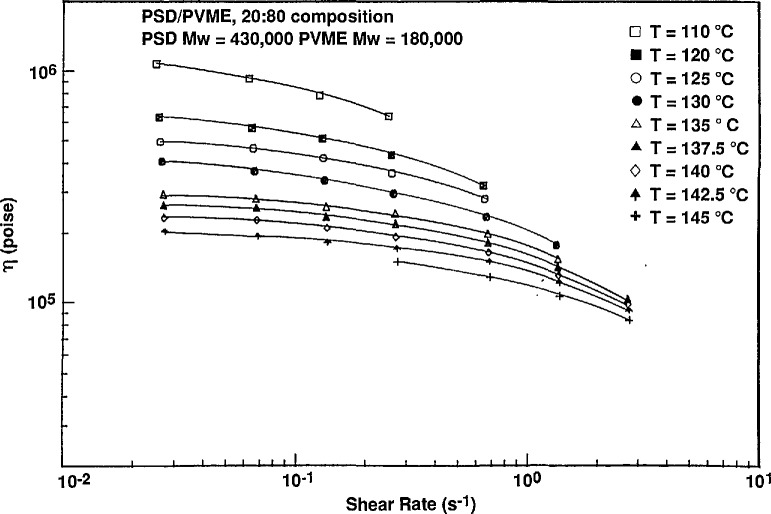
Steady shear viscosity for 20:80 PSD/PVME blend as a function of temperature as measured in the SANS shear cell.

The 440K PSD/180K PVME blend was studied between 110 °C and 145 °C at shear rates between 0.02 and 3.0 s^−1^. The optical cloud point at zero shear was 140 °C. Analysis of the zero shear scattering data using a plot of the square of the inverse correlation length versus reciprocal temperature [15] gives a value for the spinodal temperature of 140.6 °C. Torque measurements were obtained during the SANS measurements to give the steady shear viscosity values (fig. 3). At zero shear, the scattering behavior parallel and perpendicular to flow is identical as expected.

At constant temperature, the shear rate dependence of the scattering profiles show some significant differences. In the one phase region (below 140 °C) parallel to flow, the scattering intensity at low *q* decreases with increasing shear rate. Perpendicular to the flow direction, the scattering profiles are virtually independent of shear rate. An example of the shear rate dependence is shown in figures 4a and 4b for 137.5 °C.

**Figure 5:**
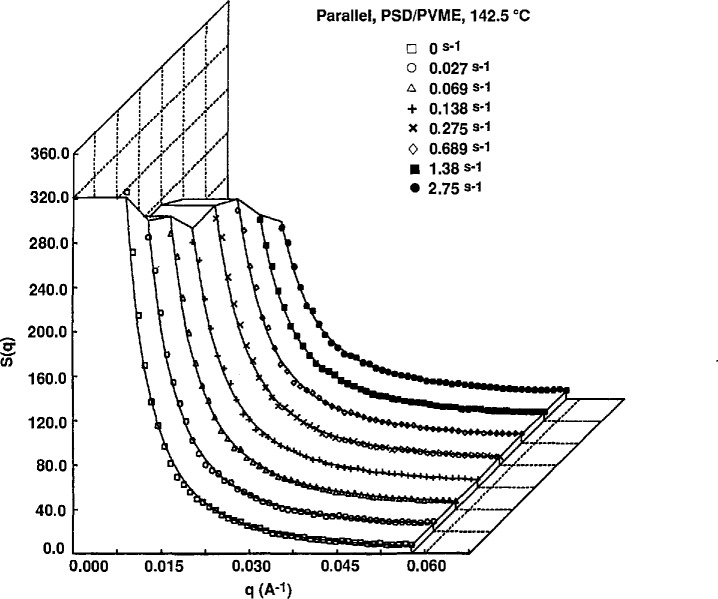
Scattering intensities as a function of shear rate for a 20:80 blend of *M*_w_ = 4.4 × 10^5^ PSD and *M*_w_ = 1.8 × 10^5^ PVME at 142.5 °C. Curves are horizontal sector averages. Solid lines represent fits to the RPA function for polymer blends of deGennes.

Above 140 °C (near phase separation), the differences in the scattering behavior are much more pronounced. The intensities at low *q* decrease with increasing shear rate in the parallel direction (fig. 5). Again, no significant changes are observed perpendicular to flow. The solid lines in the figures represent fits to the data using the expression for the structure factor of polymer blends by deGennes using the random phase approximation (RPA) [16]. The fits to the data are good except for the zero shear data in the two-phase region. With the application of shear, the RPA fits still appear reasonable even in the two-phase region. This may reflect the insensitivity of the RPA calculation of *S*(*q*) as a function of shear in a three parameter representation.

## 4. Discussion

The effects of shear on the scattering behavior of polymer blends are quite dramatic parallel to the flow direction. Use of the deGennes RPA function to fit the data may be questionable, therefore discussion on the shear rate dependence of the parameters which are typically extracted from fits to the data such as the polymer/polymer interaction parameter, χ, or the size of the concentration fluctuations, ξ, will not be attempted in this paper. On the basis of SANS studies on quiescent polymer blends [17], the decrease in the scattered intensity parallel to flow may be interpreted as an increase in the miscibility of the two polymers. In agreement with our observations, Pistoor and Binder have predicted that at high shear rates a blend of two different homopolymers would exhibit a lower scattering intensity parallel to the flow direction for simple shear [18]. Results on a polymer/polymer/solvent system using this same apparatus have also shown evidence for shear induced mixing behavior [19].

The steady shear viscosity shows no significant changes as function of temperature through the phase boundary. This is somewhat unexpected since there are many reports in the literature of unusual viscosity behavior in two phase systems [20,21]. More detailed presentations of the SANS and viscosity data and discussion will appear in a subsequent publication.

## 5. Conclusions

A device for the simultaneous study of the steady shear viscosity and small angle neutron scattering behavior of high viscosity fluids at elevated temperatures has been designed, constructed, and demonstrated to be fully operational. The effects of shear on the critical behavior of polymer blends are dramatic as evidenced by changes in the SANS behavior as a function of temperature and shear rate. The results have qualitatively been interpreted as evidence for shear induced mixing behavior in the PSD/PVME blend examined. The steady shear viscosity behavior alone was not capable of detecting the transition from one phase to two phases.

The successful application of this apparatus to the problem of shear effects in critical phenomena allows us to contemplate future experiments in the areas of single chain behavior under shear in polymer melts as well as examining the shear behavior of a variety of different materials such as liquid crystals and polyelectrolytes by small angle neutron scattering.

## Figures and Tables

**Figure 4a:**
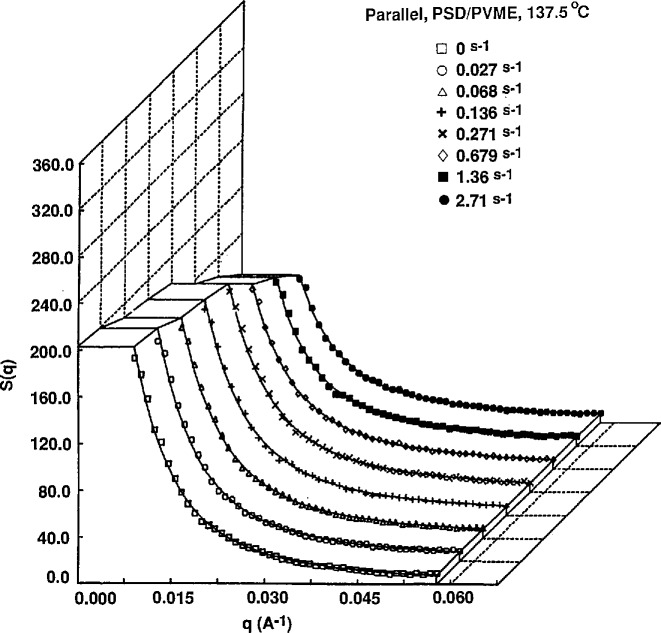
Scattering intensities as a function of shear rate for a 20:80 blend of *M*_w_ = 4.4 × 10^5^ PSD and *M*_w_ = 1.8 × 10^5^ PVME at 137.5 °C. Curves are horizontal sector averages.

**Figure 4b:**
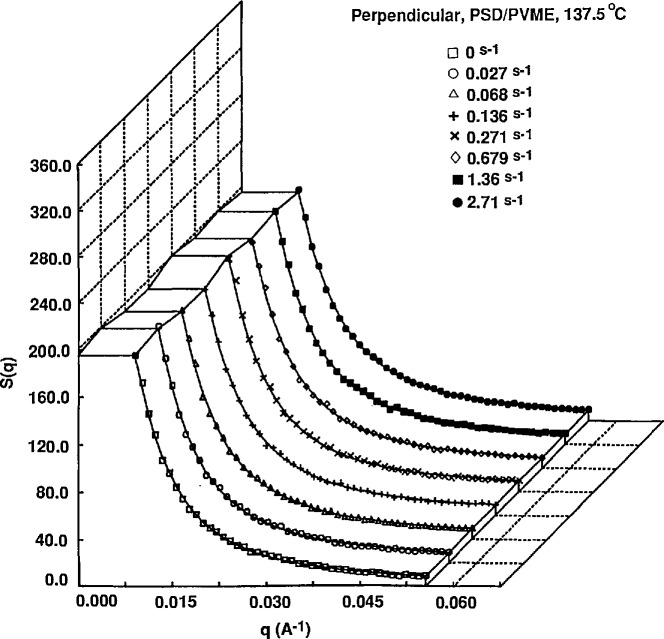
Scattering intensities as a function of shear rate for a 20:80 blend of *M*_w_ = 4.4 × 10^s^ PSD and *M*_w_ = 1.8 × 10^5^ PVME at 137.5 °C. Curves are vertical sector averages. Solid lines represent fits to the RPA function for polymer blends of deGennes.

## Footnotes

1 Certain commercial equipment, instruments, or materials are identified in this paper in order to adequately specify the experimental procedure. Such identification does not imply recommendation or endorsement by the National Institute of Standards and Technology, nor does it imply that the materials or equipment identified are necessarily the best available for the purpose.
